# Far-Red Light Acclimation for Improved Mass Cultivation of Cyanobacteria

**DOI:** 10.3390/metabo9080170

**Published:** 2019-08-19

**Authors:** Alla Silkina, Bethan Kultschar, Carole A. Llewellyn

**Affiliations:** 1 Centre for Sustainable Aquatic Research (CSAR), Bioscience department, College of Science, Swansea University, Singleton Park, Swansea SA2 8PP, UK; 2 Department of Biosciences, College of Science, Swansea University, Singleton Park, Swansea SA2 8PP, UK

**Keywords:** cyanobacteria, chromatic adaptation, LED, far-red light, growth, photosynthesis, mass cultivation, pigments, *Chlorogloeopsis*

## Abstract

Improving mass cultivation of cyanobacteria is a goal for industrial biotechnology. In this study, the mass cultivation of the thermophilic cyanobacterium *Chlorogloeopsis fritschii* was assessed for biomass production under light-emitting diode white light (LEDWL), far-red light (FRL), and combined white light and far-red light (WLFRL) adaptation. The induction of chl *f* was confirmed at 24 h after the transfer of culture from LEDWL to FRL. Using combined light (WLFRL), chl *f*, *a*, and *d*, maintained the same level of concentration in comparison to FRL conditions. However, phycocyanin and xanthophylls (echinone, caloxanthin, myxoxanthin, nostoxanthin) concentration increased 2.7–4.7 times compared to LEDWL conditions. The productivity of culture was double under WLFRL compared with LEDWL conditions. No significant changes in lipid, protein, and carbohydrate concentrations were found in the two different light conditions. The results are important for informing on optimum biomass cultivation of this species for biomass production and bioactive product development.

## 1. Introduction

Cyanobacteria are photosynthetic prokaryotes that are increasingly explored for use in industrial biotechnology. They are extremely diverse and genetically tractable, making them attractive as cell factories, and can adapt to a wide range of extreme habitats, often with the production of unique metabolites [1]. These adaptations can be exploited in industry to increase productivity and for the production of useful compounds such as pigments, mycosporine-like amino acids (MAAs), and fatty acids [2,3].

Having a long evolutionary history, cyanobacteria have evolved with the ability to cope with varying light intensities and wavelengths. They are able to modify their chlorophylls (chls) and carotenoids, as well as rearrange photosystem I (PSI), PSII, and phycobilisomes (PBS) during excess or limited light conditions [4,5]. These rearrangements allow absorption of light to maximise photosynthetic efficiencies. These light-dependent acclimation processes include; complementary chromatic acclimation (CCA), far-red light photoacclimation (FaRLiP), and low light photoacclimation (LoLiP) [6].

Chlorophyll (chl) *a* is the major photosynthetic photo-pigment within almost all organisms that utilise oxygenic photosynthesis [7]. Some cyanobacteria have photoadaptive strategies for absorbing longer wavelengths in the far-red light region (700–750 nm) by the production of chl *d* and *f* [8]. Inducible production of these chls has been seen in a variety of species, such as *Chlorogloeopsis fritschii,* PCC 6912 [9], *Synechococcus sp.* PCC 7335 [10], *Chroococcidiopsis thermalis* PCC 7203, *Leptolyngbya* sp. JSC-1, and *Calothrix sp*. PCC 7507 [4]. This phenomena, FaRLiP, achieves remodeling of PSI and PSII as well as the PBS [11], with production of chl *d*, *f*, and far-red light (FRL) absorbing phycobiliproteins to maximise photosynthesis, productivity, and survival [7,12].

*Chlorogloeopsis fritschii* (*C. fritschii*) is a subsection V cyanobacterium, first isolated from soils of paddy fields [13]. It has a variety of morphologies and is tolerant to a variety of growth conditions, which are good attributes for an industrial species [14,15]. Previous research on *C. fritschii* has shown the production of chl *d* and *f* under near infrared radiation [9].

Algal biotechnology is a developing area with continued advancements in technologies for cultivation and downstream processing. The main commercial applications of algal biomass are aquaculture feeding, bioremediation, and high value products [16]. The mass production of microalgae species, including cyanobacteria, is investigated around the world. This is because they are rich sources of bio-products such as polysaccharides, lipids, proteins, pigments, and bioactive compounds which can be utilised as feed and food and for pharmaceuticals, cosmetics, and health supplements [17]. The species-specific production of useful metabolites from the algal biomass, including cyanobacteria, has been widely reviewed for industrial biotechnological applications [3,16,17,18].

Additional research is required to understand the regulation of photosynthesis, photoprotection, and photomorphogenesis in cyanobacteria and the implication of the use of FRL in increasing productivity and/or pigment accumulation as a robust platform in industrial biotechnology [19]. In this study, we characterise the changes in productivity, pigment, and biochemical composition of *C. fritschii* during exposure to light-emitting diode white light (LEDWL) and FRL, followed by a comparison of white light with combined white light and FRL (WLFRL) results. We finish with a discussion on the application within industry.

## 2. Results

### 2.1. Growth and Productivity of C. fritschii Under LED White Light and Far Red Light

**Figure 1:**
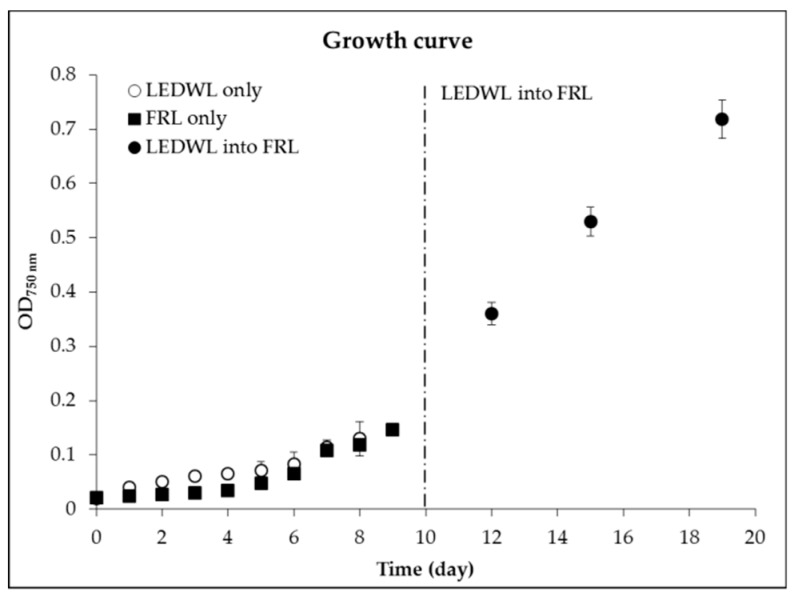
Growth curve, measured using optical density at 750 nm (OD_750 nm_) of *Chlorogloeopsis fritschii (C. fritschii)* under either light-emitting diode white light (LEDWL) or far-red light (FRL) only for 10 days. The dotted line represents the transfer of LEDWL cultures into FRL for a further 10 days.

The two growth conditions (LEDWL and FRL) showed similar growth patterns over the 9 days. Cultures grew in lag phase for the first 4 days in both conditions, followed by an exponential growth phase for 5 consecutive days (Figure 1).

An overall average growth rate (8 days, 0 to 8) for LEDWL was 0.32 (STDEV = 0.01), in comparison with FRL conditions, which provided an average growth rate of 0.26 (STDEV = 0.02), showing a low light adaptation of *C. fritschii* cultures. No significant difference was found for the accumulation of biomass during the first 8 days under the two light conditions (*p* > 0.05). From day 4, both cultures reached an exponential growth rate. After 10 days of growth under LEDWL, the cultures were transferred from LEDWL to FRL conditions, these cultures were then exposed to FRL for a further 10 days. The average growth rate of this period was 0.27. The growth of *C. fritschii* continued in the exponential phase, with a reduced rate compared to LEDWL.

The final biomass productivity of the culture under LEDWL was 0.014 g L^−1^d^−1^ (STDEV = 0.001) and 0.03 g L^−1^d^−1^ (STDEV = 0.001) for WLFRL conditions.

**Figure 2:**
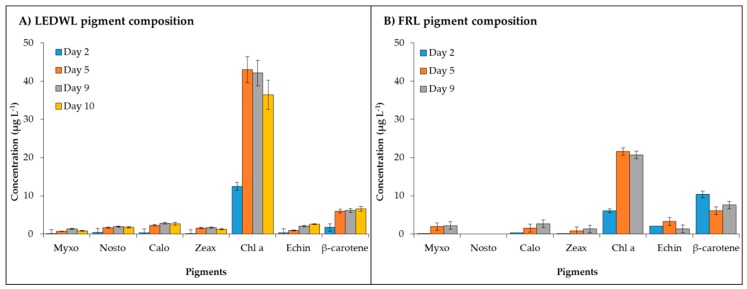
Pigment composition of *C. fritschii* under (**A**) LED white light (LEDWL) and (**B**) far red light (FRL).

The pigment profile for LEDWL showed the presence of the following pigments: Myxol-quinovoside (myxo), nostoxanthin (nosto), caloxanthin (calo), zeaxanthin (zeax) and echinenone (echin), chl *a*, and β-carotene (Figure 2A). The FRL culture had a similar pigment profile with the exception of the absence of nosto. This could be due to the concentration of this pigment below detection level of the HPLC system. A general trend of accumulation was observed for myxo, nosto, calo, and zeax under LEDWL conditions. Under FRL, the biggest changes were observed for myxo, echin, and β-carotene (Figure 2B).

Maximum concentration of chl *a* was measured for both light conditions and the cultures grown under LEDWL had double the chl *a* concentration compared to the cultures exposed to FRL. After transferring the cultures from LEDWL to FRL conditions on day 10, the cultures showed a slight decrease in chl *a* concentration. The carotenoids maintained a consistent concentration after the transfer (day 10, Figure 2A).

The final concentration of pigments on day 9 (Table 1) showed a general trend of higher concentration (µg g^−1^ of dry weight) under LEDWL compared to FRL conditions, except for myxo during FRL conditions, which showed a 1.2 times higher concentration compared to LEDWL. Other pigments, such as calo, zeax, and β-carotene, were 1.3–1.6 times higher in LEDWL cultures with chl *a* and echin concentration was over two-fold higher in LEDWL than FRL cultures (Table 1).

**Figure 3:**
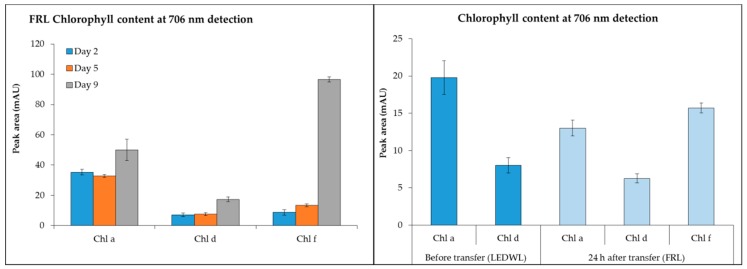
Chlorophyll content (chl *a*, chl *d*, and chl *f,* detected at 706 nm) of *C. fritschii* exposed to FRL at day 2, 5, and 9 and LEDWL (day 9) and 24 h after transfer into FRL (day 10).

−Chl *d* (detected at 706 nm) was present under both LEDWL and when transferred to FRL conditions (Figure 3) and increased gradually over time. For chl *f*, under FRL there was a ~10-fold increase at day 9 compared to day 2. After transfer of LEDWL exposed cultures to FRL, chl *f* was induced and there was a slight reduction in chl *a* and chl *d* (Figure 3).

### 2.2. Enhancement of Growth by Combining Two Light Sources (White LED Supplemented with Far Red-Light)

**Figure 4:**
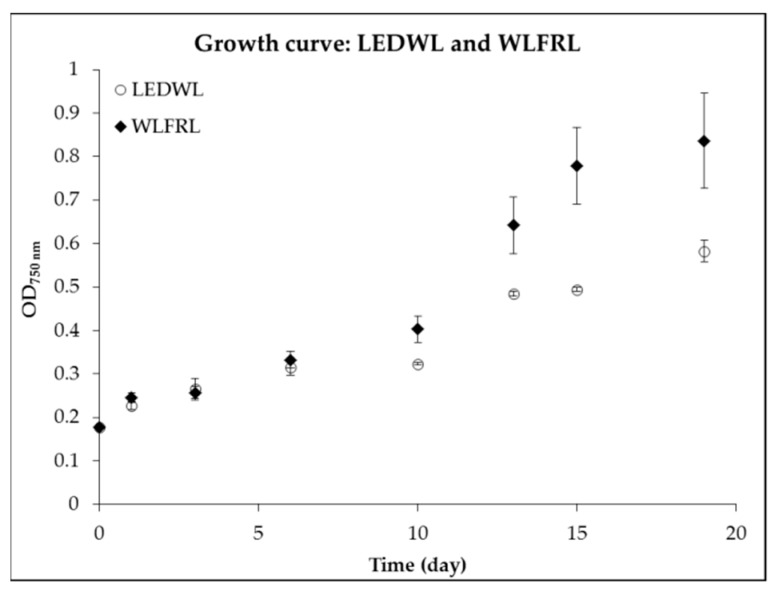
Growth curve of *C. fritschii* under LED white light (LEDWL) and supplemented LED white light with far-red light (WLFRL).

Next, the combination of LEDWL supplemented with FRL compared to LEDWL was investigated. During the first 6 days, the growth under the two light conditions (Figure 4, LEDWL and WLFRL) showed no significant difference (*p* > 0.05). After day 8, the cultures showed a difference in growth performance, with improved results for LEDWL supplemented with FRL (WLFRL). This result was shown in the average growth rate (µ) of 0.39 d^−1^ (STDEV = 0.02) for WLFRL and 0.32 d^−1^ (STDEV = 0.01) for LEDWL growth conditions (STDEV = 0.01). The exponential growth phase for WLFRL was observed over 8 days (day 8 to day 16, µ = 0.42, STDEV = 0.02), whereas the LEDWL condition had a 5 day exponential growth phase (µ = 0.33)**.** The WLFRL light combination resulted in improved growth.

**Figure 5:**
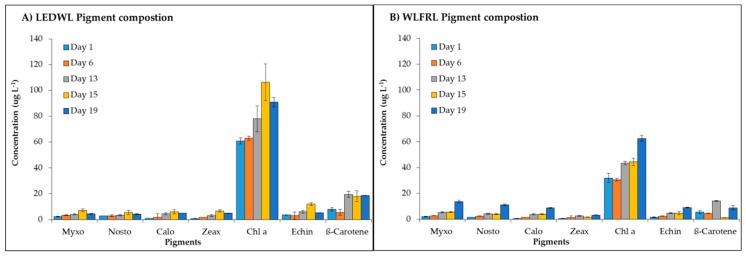
Pigment composition of *C. fritschii* exposed to (**A**) LEDWL only and (**B**) LEDWL supplemented with FRL (WLFRL).

The algal pigments, such as xanthophylls, carotenes, and chlorophylls were detected in both culture conditions (Figure 5). The WLFRL resulted in improved pigment accumulation (Figure 5B) with all pigments considerably increased in their quantity up to the last day of cultivation (day 19). During the exponential growth phase (WLFRL, day 8 to 16), the highest concentration for most of the analysed pigments was observed. The pigments under LEDWL conditions (Figure 5A) showed saturation at day 15, with a slight reduction in concentration by the final day of cultivation (day 19). Final pigment concentrations at day 19 (Table 2) showed an increase in levels under WLFRL conditions compared to LEDWL, with the exception of β-carotene, which showed increased levels in cultures exposed to LEDWL only.

**Figure 6:**
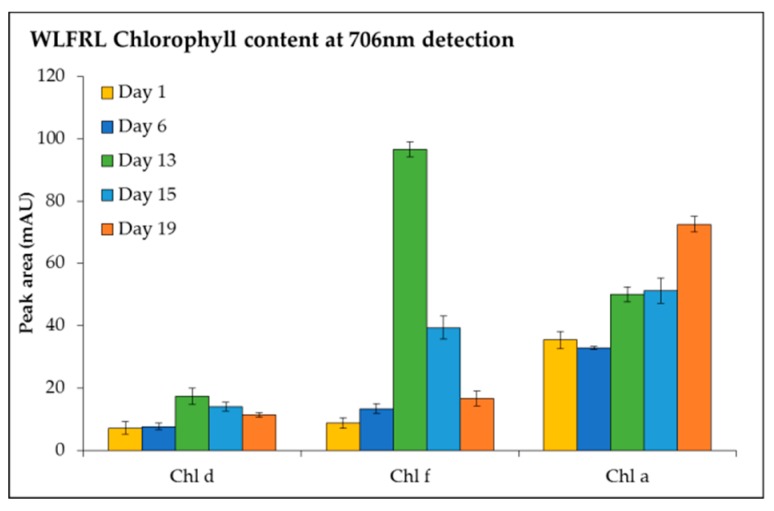
Chlorophyll content (chl *d*, chl *f*, and chl *a*, detected at 706 nm) of *C. fritschii* exposed to LED white light supplemented with far-red light conditions (WLFRL) at day 1, 6, 13, 15, and 19.

The detection of chl *f*, chl *d*, and chl *a* at 706 nm (Figure 6) was investigated under LEDWL supplemented with FRL. An increase in chl *a*, chl *d*, and chl *f* was observed for the cultures grown under supplemented far-red light (WLFRL). Chlorophyll *f* reached its maximum concentration on day 13, after which it gradually reduced to its lowest content at day 19. The same result was observed for chl *d*, with accumulation at day 13; however, the concentration was 5 times less than chl *f*. Chlorophyll *a* consistently increased during the cultivation period and by day 19 reached its maximum concentration (Figure 6).

### 2.3. Phycocyanin Concentration During LEDWL and WLFRL Conditions

**Figure 7:**
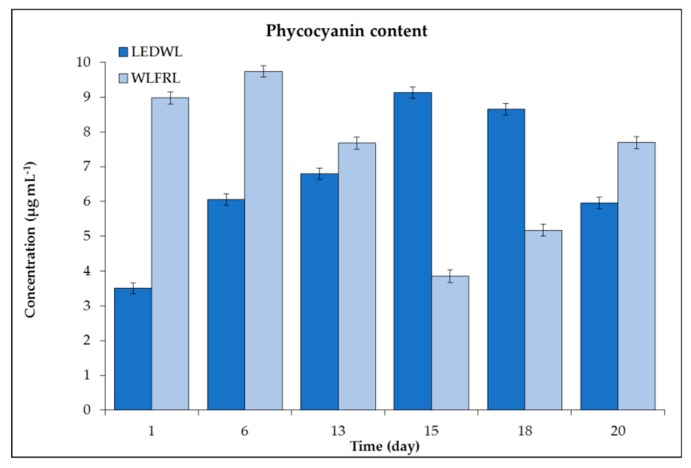
Phycocyanin concentration (µg mL^−1^) of *C. fritschii* under LEDWL and WLFRL conditions.

Cultures grown under LEDWL had high initial concentrations of phycocyanin followed by a reduction of the concentration until day 15. After this, a slight increase in the concentration was observed up to the final day of cultivation (day 20, Figure 7).

The growth conditions under WLFRL had a positive influence on the accumulation of phycocyanin. The concentration increased two-fold in two weeks of cultivation. Maximum concentrations were observed at day 15 and a slight decrease followed until day 20. The maximum concentration observed under the LEDWL and WLFRL was similar at ~9.7–9.7 µg mL^−1^. At day 13, both cultures, grown on two light conditions (LEDWL and WLFRL), revealed similar concentrations of phycocyanin, thus showing 13 days as an optimum time for the adaptation under both light regimes (Figure 7).

### 2.4. Biochemical Composition during LEDWL and WLFRL Conditions

**Figure 8:**
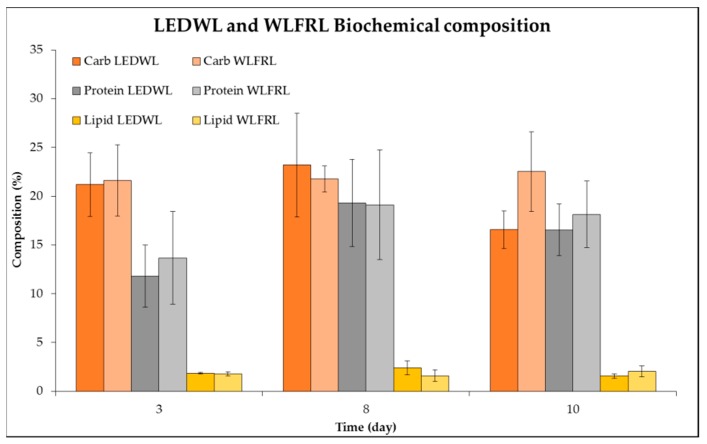
Biochemistry composition (%) of *C. fritschii* grown under LEDWL and WLFRL conditions.

Finally, the protein, carbohydrate, and lipid composition of cultures grown under two light conditions (LEDWL and WLFRL) were evaluated (Figure 8). The biomass grown under both light conditions contained 21–25% carbohydrates, 15–22% proteins, and 2–4% lipids. Statistical results (supplementary materials Table S1) showed that the light, time, and combination of both variables (light and time) did not show any significant differences.

## 3. Discussion

The discovery of chl *d* and chl *f* in terrestrial cyanobacteria demonstrated that the wavelength range of cyanobacterial photosynthesis could be extend into the far-red region (λ = 700 to ~800nm) [7,12]. This specific adaptation helps cyanobacteria utilise FRL for growth and photosynthesis [20,21]. It is clear that under FRL, cyanobacteria tend to change their metabolism and perform effective growth and active photosynthesis via metabolomics changes with the development of chl *f* and *d* as accessory pigments in antennae systems. These pigments absorb energy and transfer it to the photosynthetic reactor center (RC). Usually these pigments are not involved in the photosynthetic electron transport chain. Additionally, chl *d* as well as chl *a* can function in the photosynthetic RC [22]. Such a transformation in cyanobacterial metabolism increases the possibilities for absorbing light in longer shifted wavelengths, which is important in cyanobacterial survival. There are many studies on these unique chls, however their full function and role in cyanobacterial metabolism is still not clear, specifically how the growth and productivity will be affected for the mass cultivation of this species for biotechnological purposes.

In our research study, we can confirm that the changes in *C. fritschii* pigment composition (xanthophylls, chlorophylls, and phycocyanin) under FRL combined with LEDWL improved growth and twice increased the biomass productivity in comparison of LEDWL. This FaRLiP process triggered antennal transfer of energy to the photosynthetic RC, confirmed by an increase in chl *f* and *d* concentrations [4,22]. The combination of lights (WLFRL) changed the carotenoids’ profile. Under WLFRL, we observed an increased concentration of myxo, nosto, calo, and echin (Table 2). In comparison with mono light adaptation (LEDWL only compared to FRL only), this effect was not seen. An extensive study of carotenoid changes in cyanobacteria by Zakar et al., 2016 [23], confirmed that these pigments are responsible for the light harvesting and photoprotective capacities, showing their essential roles in photosynthetic metabolism [24,25]. The photoprotective mechanism can also occur by cyanobacterial carotenoid-proteins. One of these protein complexes is the orange carotenoid protein (OCP), discovered by David Krogmann [23,26]. The carotenoid composition of OCP is presented by 60% echin, 30% keto-carotenoid 3′hydroechninone, and 10% zeax [27]. The increase in echin and zeax under LEDWL and FRL in our study confirmed the activation of this protein and prevented cellular damage from excessive light. This effect has additionally been confirmed by non-photochemical quenching of the carotenoid-binding protein [28].

The combination of both lights activated different acclimation mechanisms and effective light assimilation for productive photosynthetic efficiency. Two main processes are involved in light adaptation, these are light energy harvest and light energy transfer [6]. In our case, by using both lights, it increased the effectiveness of both light adaptive mechanisms. The light energy harvest was demonstrated by appearance of chl *f*, chl *d,* an increased concentration of carotenoids, and phycocyanin. The light energy transfer was proved by an increased concentration of carotenoids of OCP under WLFRL in comparison with LEDWL conditions. Furthermore, the chl *d* was involved in both processes [29]. This dual mechanism of chl *d* functioning was confirmed by a recent study of transcriptional profiling of *C. fritschii* in FRL for chl *d* synthesis regulation [22]. In summary, we supposed that LEDWL maintained stable growth and that FRL activated the synthesis of chl *d* and chl *f* and restructured the functioning of PSI and PSII [11]. The combination of both lights increased growth, productivity, and oxygenic metabolism within *C. fritschii*.

Mass cultivation of *C. fritschii* is very relevant for applications in biotechnology [30]. The biology of this species has great potential for scale-up and mass cultivation in different latitudes around the world. This thermophilic cyanobacterium has several advantages in terms of large-scale growth. It requires high temperatures, which gives real advantages over other species for mass cultivation. In our study we grew this species under 25 °C, however successful growth in mass scale was shown in Balasundaram et al., 2012, and it can grow at up to 50 °C [31]. The mass cultivation set-up (PBR and raceways) could be placed in desert conditions. It has been confirmed that this species could grow on elevated CO_2_ concentrations up to 5% of CO_2_ [31,32]. It can therefore be co-located with industries emitting flue gas, e.g., power plant stations [31]. Additionally, this species could be cultivated in African and South East Asian weather conditions, as the biomass contains many valuable compounds for food, feed additivities, and as a whole food and can be used to combat malnutrition [33,34]. The application of this species as a feed for tilapia has been studied, showing that this species has potential in aquaculture [35].

Several advantages of the mass cultivation of this species are related to the aspect of easy downstream processing. This species is auto flocculating and does not require expensive equipment of membrane filtration and/or centrifugation to obtain the algal biomass paste for future processing and preservation [15,16]. This is another aspect of the development of successful mass cultivation of this species in different locations around the world, making this species a model for worldwide application.

The use of mass cultivation of *C. fritschii* in bioeconomy is an important target of algal biotechnology. The understanding of their cell physiology and specific light adaptation will help to improve the biomass and specific compounds production. The main bioactive compounds of *C. fritschii* are presented in Table S2. The principle groups are mycosporine-like amino-acids (MAAs) and pigments. *Chlorogloeopsis* produces chlorophylls, carotenoids, and phycobiliproteins, which contain different colours and can be used as biodegradable dyes [15,36]. Furthermore, bioproducts such as biodegradables and biocompatible plastic could be produced by *Chlorogloeopsis*. Nowadays, these are very important biomolecules, with the potential to be used as a substitute for single use plastic. The reason for this is that petrochemical and non-biodegradable contamination presents a major problem worldwide [37].

Many other applications of *Chlorogloeopsis* and cyanobacteria could be developed. This algal group can produce antimicrobial, antiviral, anticancer, and antiprotozoal compounds for pharmaceutical applications and can be used as a food, feed, and in other value-added products [38]. Further research and product development activities need to be established. In our research, we confirmed that the production of the main group of pigments (chlorophylls, carotenes, xanthophylls, and phycocyanin) could be of potential commercial interest.

## 4. Conclusions

A combination of LEDWL and FRL showed higher productivity of *C. fritschii*, with an increased concentration of myxo, nosto, calo, and echin. These combined light conditions triggered light harvesting and light energy transfer together with the induction of chls *d* and *f*, giving increased growth, photosynthetic effectiveness, and double the productivity of *C. fritschii* cultures. However, the overall protein, lipid, and carbohydrate composition did not significantly change under WLFRL. Our results suggested that the overall production of this biotechnologically promising species can be increased by cultivation using additional far-red light.

## 5. Materials and Methods

### 5.1. Experimental Design

For the first experiment, three flasks with a total volume of 800 mL each were placed under FRL and LEDWL conditions. The initial cell concentration was 0.5 × 10^6^ cell mL^−1^ (or 750 nm measurements ~0.05).

On day 9, at an OD_750_ of 0.3–0.4, the flasks under FRL were harvested. The cultures grown under LEDWL conditions were sampled in triplicate for growth and pigment analysis and then transferred to FRL (far red light) conditions for a further 6 days. After 24 h of exposure to FRL, the cultures were sampled again in triplicate for pigments and growth analysis.

For the second experiment, three flasks with a total volume of 800 mL of *C. fritschii* culture were placed under LEDWL and FRL combined with LEDWL. The initial cell concentration was 2 × 10^6^ cell mL^−1^ (or 750 nm measurements ~0.2). The duration of the experiment was 20 days.

Every 24 h, the growth parameters, such as cell concentration, biovolume, and OD (optical density) was measured and a collection of the algal biomass (harvested from 15 mL of culture) was made for pigment analysis by HPLC, spectrophotometer, and for biochemical analysis by FTIR.

### 5.2. Source Organism and Cultivation Conditions

*Chlorogloeopsis fritschii* was purchased from Pastor Culture Collection (PCC 6912; Paris, France). The master axenic culture was maintained in a temperature and white fluorescent light-controlled room (T = 28 °C) with 16:8 h light: dark cycle.

### 5.3. Growth Estimation

Every 24 h cell concentration and biovolume by Coulter counter (Multisizer 4, Beckman, USA) measurement was performed to quantify culture growth. Further details are described in Reference [39]. The OD (750 nm) measurements were analysed by UNICAM UV 300 spectrophotometer.

During sampling days, a minimum of 50 mL of culture was taken from each tube and centrifuged (Beckman Coulter Centrifuge, Avanti J-20XP) for 20 min at 8000 rpm. The biomass was washed twice with deionized (DI) water, centrifuged for 20 min at 8000 rpm, then collected and freeze dried (ScanVac Cool Safe, LaboGene, Lynge, Denmark) for 24 h prior to further analysis.

The specific growth rate (μ) was determined for all LED light treatments using Equation (1), where *N*_0_ and *N*_1_ are the cell concentrations (cells mL^−1^) at times *t*_0_ and *t*_1_, as follows:

µ = lnN_1_ − lnN_0_/*t*_1_ − *t*_0_. (1)

### 5.4. Dry Weight and Biomass Productivity

Dry weight was measured according to Reference [39]. A known volume of algae was pelleted and washed with deionized (DI) water (three times using 25 mL of DI water each time) prior to being filtered onto pre-weighed and dried filters (Whatman GF/F 47 mm Ø). The filters with algal biomass were then dried and re-weighed until constant weight was reached. Dry weight (g L^−1^) was then calculated by subtraction of the final filter weight and the pre-filtered weight.

Biomass productivity was calculated as the difference in terms of DW between the sample day and the previous day. The results are expressed in g L^−1^ d^−1^.

### 5.5. Pigments Extraction and Measurements

A known mass of frozen cell paste was transferred to an extraction tube containing 1 mL HPLC grade acetone and 0.2 mg zirconium (0.1 mm diameter) beads. The sample was then lysed in Precellys^®^24, a high-throughput tissue homogenizer, at 6500 rpm 2 × 30 s with a pause of 5 s. The sample was centrifuged (5 min at 20,000× *g*, Microcentrifuge) and the removed supernatant was used for pigment analysis on HPLC (Agilent HP 1200).

### 5.6. HPLC

The pigment extract was analysed using a high performance liquid chromatography (HPLC) method described previously (Method C in Airs et al., 2001 [40]). Pigment extracts were injected (100 μL) onto the HPLC column (2 Waters Spherisorb ODS2 cartridges coupled, each 150 × 4.6 mm, particle size 3 μm, protected with a precolumn containing the same phase). Elution was carried out using a mobile phase comprising methanol, acetonitrile, ammonium acetate (0.01 M), and ethyl acetate (Method C in Airs et al. 2001) at a flow rate of 0.7 mL min^−1^. The photodiode array PDA detector was set to monitor wavelengths at 406, 440, 660, 696, and 706 nm. Carotenoids and chl-*a* were quantified against standards (Sigma) and, for chls *d* and *f*, peak areas were used [7,9].

### 5.7. Phycocyanin Extraction and Determination

Phycocyanin (PC) was extracted using a modified version [41] of the method developed by Reference [42]. The freeze-dried biomass of each sample was weighed to a known weight on a semi-micro and analytical balance (MSE 124S-100-DU, Sartorius balance, Germany). The sample weight was noted to the nearest 0.1 mg and all PC extractions were conducted in triplicate. The samples were transferred into 15 mL falcon tubes and subjected to a minimum of five freeze-thaw cycles; the samples were immersed in 5 mL of 0.1 mol L^−1^ phosphate buffer (pH = 6) and stored at −20 °C until frozen (∼2 h), they were then thawed and subjected to 10 min sonication on ice [43]. The samples were then vortexed for 5 min and then placed back into −20 °C, and the process repeated. After the final freeze-thaw cycle, the cell debris was removed via centrifugation at 8000 rpm for 5 min. The supernatant was recovered and used for PC measurements. Absorbance of the extracts was measured at 592, 618, and 645 nm using a UNICAM UV 300 spectrophotometer. The concentration of the PC was determined using the equations in Reference [44], where OD is the optical density of the pigment at the particular wavelength.

PC (mg mL^−1^) = [(OD618 nm − OD645 nm) − (OD592 nm − OD645 nm) × 0.15] × 0.15 (2)

### 5.8. Proteins, Lipids, and Carbohydrate Analysis Using Fourier Transformed Infra-Red (FTIR)

FTIR attenuated total reflectance (ATR) spectra were collected using a PerkinElmer Model Spectrum Two instrument equipped with a diamond crystal ATR reflectance cell with a DTGS detector scanning over the wavenumber range of 4000–450 cm^−1^ at a resolution of 4 cm^−1^ as described by References [45,46]. Briefly, ethanol (70%) was used to clean the diamond ATR before the first use and between samples. Approximately 3–5 mg of finely powdered freeze-dried *C. fritschii* biomass was applied to the surface of the crystal and then pressed onto the crystal head. A duplicate (each consisting of an average of 12 scans) of each bioreactor sample was conducted for each light type; therefore, results of 6 ATR spectra were gained and the results were averaged. Background correction scans of ambient air were made prior to each sample scan. Scans were recorded using the spectroscopic software Spectrum (version 10., PerkinElmer, Germany). The contents of lipids, proteins, and carbohydrates in the biomass samples were determined using FTIR, which had previously been calibrated using mix of monosugars (rhamnose, xylose, glucuronic acid, and glucose) for carbohydrates, palmitic acid for lipids, and BSA for proteins at different concentrations. The carrier powder for the FTIR calibration was potassium bromide (KBr) [41].

### 5.9. Statistical Analysis

Statistical analyses were carried out using the R project software on the OD, pigments concentration, lipids, carbohydrates, and protein content data. Data normality was tested using a Shapiro test. Non-normal data significance was assessed using GLMs (generalised linear models) furthered by an analysis of variance (ANOVA) on a data set not following a normal distribution. Crossed factor ANOVAS were carried out on normally distributed data. Both statistical methods tested the impact of experimental duration, pigments and intracellular lipids, carbohydrates, and protein content. When statistical significance was found, post hoc Tukey tests were implemented.

All the experiments were performed in triplicate. The standard deviation and means were analysed for significance using the biostatistics software Excel through one-way ANOVA. The Duncan multiple range test was used to compare the significance of difference among tested algae at *p* values of < 0.05. Results are reported as ± SD or error bars.

## Figures and Tables

**Table 1:** Concentration (µg g^−1^ of dry weight) of main pigments present at day 9 within *C. fritschii* biomass during LEDWL and FRL conditions, including fold change comparing LEDWL and FRL.

Pigments	LEDWL	FRL	Fold Change
Myxo	3.9 ±.0.3	4.9 ± 0.8 *	1.3
Nosto	5.6 ±0.5	N/A	N/A
Calo	8.3 ± 0.8	6.1±0.8 **	0.7
Zeax	4.8 ± 0.6	2.9±0.6 **	0.6
Chl a	124.7 ± 5.4	47.6±1.6 ***	0.4
Echin	6.1 ± 0.8	3.5±0.6 **	0.6
β-carotene	18.35 ± 1.4	17.2±1.8 *	0.9

Statistical significance was measured using a two-sample t-Test with equal variance, * = 0.05 ≤ *p* > 0.01, ** = 0.01 ≤ *p* > 0.001, *** = *p* ≤ 0.001.

**Table 2:** Final pigment concentration (µg g^−1^ of dry weight) of main pigments present in *C. fritschii* biomass at day 19 of experiment trial for LEDWL and WLFRL conditions, including fold change comparing LEDWL and WLFRL.

Pigments	LEDWL	WLFRL	Fold Change
Myxo	13.4 ± 1.9	63.6 ± 6.23 ***	4.7
Nosto	12.5 ± 1.1	52.2 ± 4.9 ***	4.2
Calo	15.1 ± 1.9	41.7 ± 2.9 ***	2.8
Zeax	14.8 ± 1.2	15.5 ± 1.2	1.0
Chl a	275.4 ± 12.5	288.5 ± 25.8 *	1.0
Echin	15.7 ± 1.2	41.7 ± 1.6 **	2.7
β-Carotene	56.2 ± 2.7	41.1 ± 1.6 *	0.7

Statistical significance was measured using a two-sample t-Test with equal variance, * = 0.05 ≤ *p* > 0.01, ** = 0.01 ≤ *p* > 0.001, *** = *p* ≤ 0.001.

## Supplementary Materials

The following are available online at https://www.mdpi.com/2218-1989/9/8/170/s1, Table S1: Statistical analysis of composition of *C. fritschii* culture, Table S2: Bioactive compounds produced by *Chlorogloeopsis* sp.
