# Supplementary material for: Far-Red Light Acclimation for Improved Mass Cultivation of Cyanobacteria
Source: Metabolites. 2019 Aug 19;9(8):170. doi: 10.3390/metabo9080170 (PMC6724174; doi:10.3390/metabo9080170)
Supplement: Supplementary file 1 [file metabolites-09-00170-s001.pdf]

rreid@wustl.eduSupplementary Materials:

Table 1. Statistical analysis of composition of *C. fritschii* culture.

Analysis	Carbohydrates	Lipids	Proteins
**Variable 1**	Day: **p= 0.373 ns**	Day: **p= 0.742 ns**	Day: **p= 0.0503 ns**
**Variable 2**	Light: **p= 0.340 ns**	Light: **p= 0.540 ns**	Light: **p= 0.5839 ns**
**Variable 3**	Day*Light: **p= 0.211 ns**	Day*Light: **p= 0.106 ns**	Day*Light: **p= 0.8976 ns**
**Statistical conclusion**	No significant differences: carbohydrates, lipids and proteins content is not different between treatments nor between days.		

Table 2. Bioactive compounds produced by *Chlorogloeopsis* sp.

Products	Compound	Use	Reference
Photo protective compounds	Mycosporine-like amino-acids (MAA)	Sun-screen protectant	[1–3]
	Scytonemin	Sunscreen pigment	[4]
Pigments	Chlorophyll *a*	Pharmaceutical and cosmetic (deodorant)	[5]
	Phycocyanin	Food colorant (ice cream, sweets) cosmetics, biomedicine: immunofluorescent techniques, antibody labelling	[6]
	Zeaxanthin	Use as a compound in treatment of age-related macular degeneration, anticancer activity	[7]
Bioplastics	Polyesters of polyhydroxy alkanoates (PHA)	Thermoplastic comparable to the synthetic plastic, polypropylene	[8]
	Polyhydroxybutyrate (PHB)	Highly hydrophobic, thermoplastic, biodegradable and biocompatible	[8,9]
Plant growth promoters	Cytokinin	Promotes cell division in plant roots and shoots	[10]

References

1. Chrapusta, E.; Kaminski, A.; Duchnik, K.; Bober, B.; Adamski, M.; Bialczyk, J. Mycosporine-Like Amino Acids: Potential Health and Beauty Ingredients. *Mar. Drugs* **2017**, *15* (10), 1–29. https://doi.org/10.3390/md15100326.
2. Kultschar, B.; Dudley, E.; Wilson, S.; Llewellyn, C. A. Intracellular and Extracellular Metabolites from the Cyanobacterium Chlorogloeopsis Fritschii, PCC 6912, During 48 Hours of UV-B Exposure. *Metabolites* **2019**, *9* (4), 74. https://doi.org/10.3390/metabo9040074.
3. Derikvand, P.; Llewellyn, C. a.; Purton, S. Cyanobacterial Metabolites as a Source of Sunscreens and Moisturizers: A Comparison with Current Synthetic Compounds. *Eur. J. Phycol.* **2017**, *52* (1), 43–56. https://doi.org/10.1080/09670262.2016.1214882.
4. Zhang, G.; Zhang, Z.; Liu, Z. Scytonemin Inhibits Cell Proliferation and Arrests Cell Cycle through Downregulating Plk1 Activity in Multiple Myeloma Cells. *Tumor Biol.* **2013**, *34* (4), 2241–2247. https://doi.org/10.1007/s13277-013-0764-5.
5. Da Silva Ferreira, V.; Sant'Anna, C. Impact of Culture Conditions on the Chlorophyll Content of Microalgae for Biotechnological Applications. *World J. Microbiol. Biotechnol.* **2017**, *33* (1), 1–8. https://doi.org/10.1007/s11274-016-2181-6.
6. Tavanandi, H. A.; Mittal, R.; Chandrasekhar, J.; Raghavarao, K. S. M. S. Simple and Efficient

Method for Extraction of C-Phycocyanin from Dry Biomass of Arthospira Platensis. *Algal Res.* **2018**, *31* (August 2017), 239–251. https://doi.org/10.1016/j.algal.2018.02.008.

7. Zuluaga, M.; Gueguen, V.; Pavon-Djavid, G.; Letourneur, D. Carotenoids from Microalgae to Block Oxidative Stress. *BioImpacts* **2017**, *7* (1), 1–3. https://doi.org/10.15171/bi.2017.01.
8. Balaji, S.; Gopi, K.; Muthuvelan, B. A Review on Production of Poly β Hydroxybutyrates from Cyanobacteria for the Production of Bio Plastics. *Algal Res.* **2013**, *2* (3), 278–285. https://doi.org/10.1016/j.algal.2013.03.002.
9. Hein, S.; Steinbüchel, A.; Hai, T. Multiple Evidence for Widespread and General Occurrence of Type-III PHA Synthases in Cyanobacteria and Molecular Characterization of the PHA Synthases from Two Thermophilic Cyanobacteria: Chlorogloeopsis Fritschii PCC 6912 and Synechococcus Sp. Strain MA1. *Microbiology* **2015**, *147* (11), 3047–3060. https://doi.org/10.1099/00221287-147-11-3047.
10. Singh, J. S.; Kumar, A.; Rai, A. N.; Singh, D. P. Cyanobacteria: A Precious Bio-Resource in Agriculture, Ecosystem, and Environmental Sustainability. *Front. Microbiol.* **2016**, *7* (APR), 1–19. https://doi.org/10.3389/fmicb.2016.00529.
